# Complete Response to Selective RET Inhibition With Selpercatinib (LOXO-292) in a Patient With *RET* Fusion–Positive Breast Cancer

**DOI:** 10.1200/PO.20.00282

**Published:** 2021-01-11

**Authors:** Satomi Watanabe, Masayuki Takeda, Tomoyuki Otani, Takeshi Yoshida, Kazuko Sakai, Elizabeth Olek, S. Michael Rothenberg, Jennifer Kherani, Pearl P. French, Kazuto Nishio, Akihiko Ito, Kazuhiko Nakagawa

**Affiliations:** ^1^ Department of Medical Oncology, Kindai University Faculty of Medicine, Osaka-Sayama, Osaka, Japan; ^2^ Department of Pathology, Kindai University Faculty of Medicine, Osaka-Sayama, Osaka, Japan; ^3^ Department of Genome Biology, Kindai University Faculty of Medicine, Osaka-Sayama, Osaka, Japan; ^4^ Loxo Oncology Inc., a wholly owned subsidiary of Eli Lilly and Company, Stamford, CT; ^5^ Eli Lilly and Company, Indianapolis, IN

## INTRODUCTION

Activating *RET* gene fusions have been reported in < 10% of papillary thyroid cancers and in 1%-2% of non–small-cell lung cancers.^1-7^ In a large-scale genomic profiling study, *RET* gene fusions were identified in only 16 of 9,693 (0.17%) patients with breast cancer.^8^ Selpercatinib (LOXO-292) is a highly selective and potent, CNS-penetrant RET inhibitor that has demonstrated significant antitumor activity with a tolerable safety profile in patients with solid tumors harboring diverse *RET* alterations (eg, activating gene fusions, point mutations, and indels) in the ongoing registrational LIBRETTO-001 study (ClinicalTrials.gov identifier: NCT03157128).^9,10^ These results led to the recent approval by the US Food and Drug Administration (FDA) for the treatment of metastatic *RET* fusion–positive non–small-cell lung cancer and advanced or metastatic *RET*-mutant medullary thyroid and *RET* fusion–positive thyroid cancers.^11^ Here, we describe the first patient with *RET* fusion–positive breast cancer treated with selpercatinib in LIBRETTO-001.

## CASE REPORT

**FIG 1.**
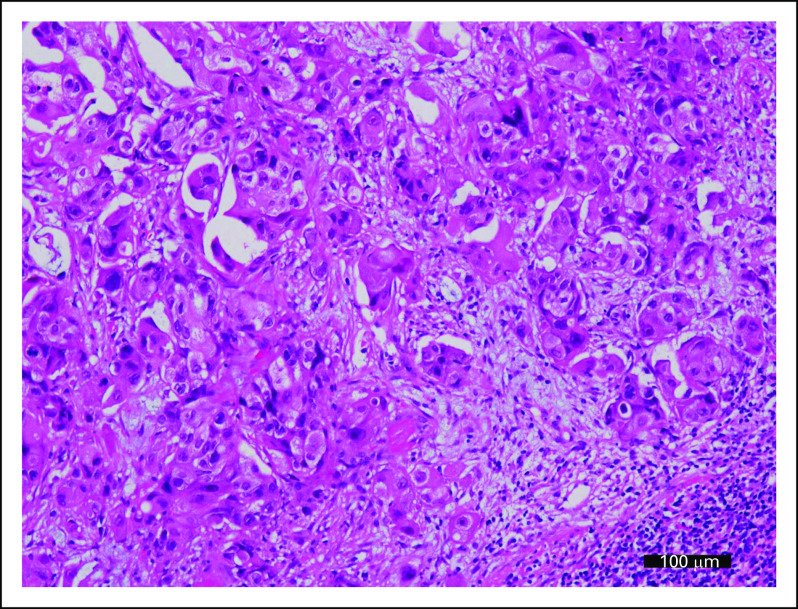
The biopsy of the right axial lymph node revealed invasive carcinoma with focal micropapillary pattern.

A 46-year-old premenopausal Japanese woman was referred to Kindai University Hospital with fluorodeoxyglucose-avid right axillary, right neck, and mediastinal lymphadenopathy on positron emission tomography-computed tomography (PET-CT) imaging on day 0. Ultrasound imaging identified a hypoechoic nodule in the right breast; right axillary lymph node fine-needle aspiration biopsy performed at previous hospital on day 13 revealed invasive carcinoma with a focal micropapillary pattern (Fig 1). Immunostaining of estrogen receptor (ER) and progesterone receptor (PgR) was evaluated by using the Allred score and the Allred scores were positive (proportion score [PS] 1 (< 1%), intensity score [IS] 2) and negative (PS 0, IS 0), respectively.^12,13^ The tumor was human epidermal growth factor 2 (HER2)–negative (immunohistochemistry [IHC] 0). Given these results, she was diagnosed with stage IV breast cancer.

**FIG 2.**
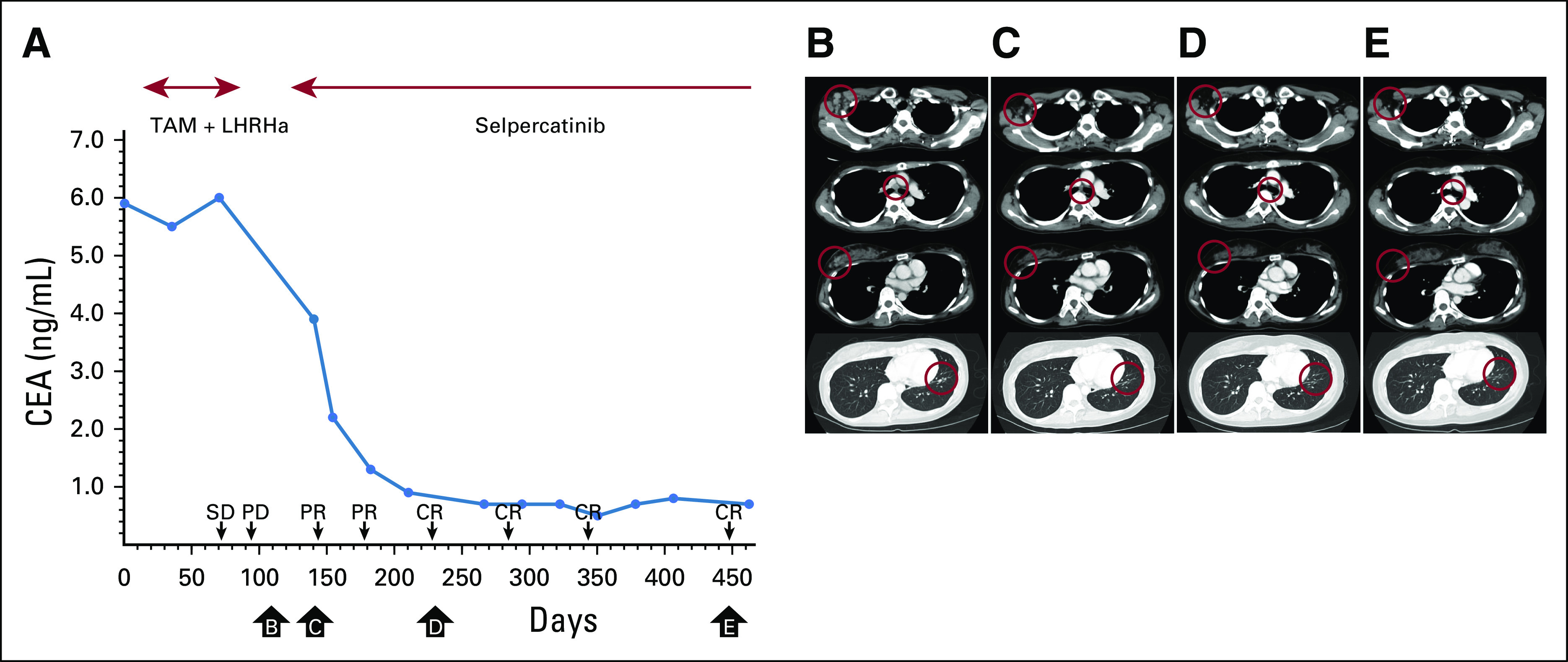
Serial monitoring of CEA and the results of response evaluation using RECIST version 1.1 (A). Each arrow (B-E) corresponds with CT imaging performed during the treatment. (B) Baseline CT scan revealed multiple right axial and mediastinal lymph node metastases, metastases in the right breast, and left lung metastases. (C) First response assessment at 21 days after treatment initiation revealed shrinkage of all the metastases, which was evaluated as partial response. (D) Repeat imaging after 3 months of the treatment showed CR. (E) The most recent CT scan revealed that the patient remains in CR. CEA, carcinoembryonic antigen; CR, complete response; CT, computed tomography; LHRHa, luteinizing hormone-releasing hormone agonist; PD, progressive disease; PR, partial response; TAM, tamoxifen.

Targeted next-generation sequencing (NGS) analysis using the FoundationOne Companion Diagnostic panel (Foundation Medicine, Cambridge, MA) was performed on the right axillary lymph node specimen. The result of NGS was reported on day 58, and the NGS identified a *CCDC6-RET* fusion (C1; R12) with no other reported genomic alterations known to contribute to human breast tumorigenesis, including none in *BRCA1* or *BRCA2*. *CTNNB1* M739I, *KEL* R14H, *MET* L211W, and *MTOR* R2110Q were detected as variants of unknown significance in the patient’s tumor. Consistent with local standard-of-care guidelines, she received treatment with tamoxifen plus goserelin from day 14 to day 91, but these were discontinued due to progression in the right breast and new lesions detected in the left lower lung. Rebiopsy of the right breast tumor revealed the following results: ER Allred score 2 (PS 1 [< 1%], IS 1), PgR Allred score 2 (PS 1, IS 1), HER2 IHC 2+, HER2 fluorescence in situ hybridization negative (HER2/HER2/CEP(centromere)17 = 0.9), and programmed death ligand 1 (SP142) expression on tumor-infiltrating immune cells of 1%-4%. On day 126, she was started on treatment with selpercatinib at the recommended phase 2 dose of 160 mg twice daily in the LIBRETTO-001 study after providing written informed consent from the patient to publish information and images. She experienced rapid clinical improvement with a resolution of right breast and neck pain and erythema. Carcinoembryonic antigen levels rapidly decreased (Fig 2A), and spiral CT imaging on day 147 demonstrated a partial response by using RECIST version 1.1 (RECIST 1.1) (overall tumor reduction −30%), with a reduction in multiple right breast masses, axillary, neck and mediastinal lymphadenopathy, and left lung metastases; follow-up CT scan repeated on day 231 revealed a complete tumor response by using RECIST 1.1 (Fig 2D). At the time of this writing, she remains in complete response and on treatment for > 300 days, with all adverse event grades 1-2 (dry skin, dry mouth, weight gain, transaminitis, and blood bilirubin increased). Most adverse events recovered to baseline with medical management, and none required dose interruption or modification.

## DISCUSSION

Selpercatinib is a first-in-class selective RET inhibitor that recently received US FDA approval for the treatment of metastatic *RET* fusion–positive non–small-cell lung cancer and advanced or metastatic *RET*-altered thyroid cancers.^9-11^ However, for patients with *RET* fusion–positive breast cancer, standard of care is currently limited to hormonal therapy, chemotherapy, and anti-HER2–targeted therapies based on hormone receptor and HER2 status.

Sorafenib and vandetanib, multikinase inhibitors with preclinical inhibitory activity against RET, have been used to treat unselected patients with breast cancer, but minimal clinical activity was observed.^14,15^ A patient with *NCOA4-RET*–positive breast cancer experienced a partial response to the multikinase inhibitor cabozantinib in combination with trastuzumab and exemestane although the cabozantinib dose was reduced for toxicity, the total time on treatment was short, and the relative contribution of each agent to the overall antitumor activity was not known.^8^ In addition, although cabozantinib has preclinical inhibitory activity against RET, its much stronger inhibition of other kinases (eg, VEGFR2) likely accounts for its clinical activity.^16,17^ In contrast, in the current case, the highly selective and potent RET inhibitor selpercatinib demonstrated a durable single-agent response in a patient with *RET* fusion–positive breast cancer.

To our knowledge, this is the first report of a breast cancer patient with a complete and sustained response to selective, *RET*-targeted therapy and adds to the diversity of *RET* fusion–positive tumor types that may benefit from selective RET inhibition. LIBRETTO-001 continues to enroll patients with *RET* fusion–positive solid tumors, including breast cancer. Additionally, broad-based genomic profiling in patients with refractory breast cancer should be considered to identify potentially actionable alterations such as *RET* gene fusions. Continued characterization of the overall frequency of *RET* fusions in breast cancer and other solid tumors is warranted.
